# Endobronchial streptokinase for airway thrombus: a case series

**DOI:** 10.1186/cc14291

**Published:** 2015-03-16

**Authors:** D Lloyd, J Bomford, M Barry, W Berry, N Barrett, L Camporota, N Ioannou, B Lams, C Langrish, C Meadows, A Retter, D Wyncoll, G Glover

**Affiliations:** 1 Guys and St Thomas' NHS Trust, London, UK

## Introduction

Pulmonary haemorrhage (PH) is common in patients receiving mechanical ventilation and especially during ECMO, due to severe lung pathology and systemic anticoagulation. Whilst PH manifests as worsening ventilation and gas exchange, in ECMO patients who already have low tidal volume and who do not rely on pulmonary gas exchange, deterioration may not be evident until extensive airway thrombus (AT) has developed. Management of AT is challenging, with lavage, suctioning, mechanical disruption and extraction of limited efficacy in severe cases. Limited reports suggest that topical thrombolytics may have a role in the management of AT [1]. We report the safety and efficacy of endobronchial streptokinase (EBSK) in patients with extensive AT.

## Methods

A retrospective case series in a UK ECMO centre. Patients who received EBSK between 2010 and 2014 were identified from pharmacy records.

## Results

Five patients were identified, 80% were male. Median age was 40 years, APACHE II score 36.5 and Murray score 3.75. Four were on ECMO with systemic heparin. All had ARDS secondary to lung infections (community-acquired pneumonia (two), lung abscess (one), TB (one) and PJP (one)). All had extensive AT, diagnosed on bronchoscopy, causing occlusion of the trachea or major bronchi, refractory to physiotherapy, lavage, suctioning ± rigid bronchoscopy. Patients received up to three administrations of EBSK, 1,000 u/ml in saline 0.9% under bronchoscopic guidance. Dose per administration was 30,000 to 80,000 u and total dose was 30,000 to 150,000 u (375 to 1,500 u/kg), with interval bronchoscopy after several hours for lavage and suctioning of lysed clot. In all cases EBSK was well tolerated with no immediate complications and no clinically significant change in systemic laboratory coagulation parameters at 12 or 24 hours compared with pretreatment baseline. In all cases, significant clearance of airway thrombus was achieved. Median tidal volume increased from 60 ml pre treatment to 170 ml at 24 hours. Median PaO_2_ during the 'FiO_2_ 1.0 test' improved from 9.0 to 17.6 kPa at 24 hours. No major bleeding, intracerebral haemorrhage or ECMO cannulae bleeding was seen up to 7 days post treatment.

## Conclusion

In this series, the largest reported to date, and the first on ECMO, EBSK was highly effective in achieving clearance of AT with subsequent improvements in pulmonary mechanics and gas exchange. No major disturbance of systemic coagulation parameters or major haemorrhagic complications occurred. The use of EBSK may be considered for refractory AT.
